# Structural changes of the multifidus in animal models of intervertebral disk degeneration: a systematic review

**DOI:** 10.3389/fsurg.2024.1482821

**Published:** 2024-12-16

**Authors:** Yaobin Wang, Xinghua Zhao, Xiangyu Zhang, Yuhua Yang, Weikang Zhang, Shaocheng Liu, Zhenlei Liu, Lei Zhang, Kai Wang, Hao Wu

**Affiliations:** ^1^ Department of Neurosurgery, Xuanwu Hospital, Capital Medical University, Beijing, China; ^2^ Department of Neurosurgery, China-Japan Union Hospital of Jilin University, Changchun, China; ^3^ Department of Critical Care Medicine, Mentougou Hospital, Beijing, China

**Keywords:** intervertebral disc degeneration, low back pain, paraspinal muscles, multifidus, animal models

## Abstract

**Study design:**

Low back pain (LBP) is a widespread clinical symptom affecting nearly all age groups and is a leading cause of disability worldwide. Degenerative changes in the spine and paraspinal tissues primarily contribute to the etiology of LBP.

**Objectives:**

We conducted this systematic review of animal models of paraspinal muscle (PSM) degeneration secondary to degenerative intervertebral disc (IVD), providing a comprehensive evaluation of PSM structural changes observed in these models at both macroscopic and microscopic levels.

**Methods:**

PubMed, EMBASE, Web of Science, Cochrane Library, and MEDLINE Ovid databases were searched through November 2023. Literature was sequentially screened based on titles, abstracts, inclusion of animal models and full texts. A manual search of reference lists from all eligible studies was also performed to identify any eligible article. Two independent reviewers screened the articles according to inclusion and exclusion criteria. The risk of bias was assessed using the Systematic Review Centre for Laboratory Animal Experimentation's Risk of Bias tool.

**Results:**

A total of nine studies were included in the final analysis after a comprehensive screening process. The included studies were assessed for various aspects of the multifidus muscle. Given the limited number of studies and the substantial heterogeneity among them, a quantitative meta-analysis was deemed inappropriate.

**Conclusions:**

This systematic review shows a comprehensive analysis of structural changes in the multifidus muscle in animal models of IVD degeneration and offers crucial insights for developing improved rodent models of IVD degeneration and assessing a battery of approaches for multifidus degeneration.

## 1 Introduction

Low back pain (LBP) is a prevalent symptom affecting almost all age groups and is the leading cause of physical disabilities worldwide, according to the 2021 Global Burden of Disease study. The incidence of LBP is relatively higher among women than men. Notably, with the aging population, the prevalence of LBP is expected to increase significantly by 2050 (1). In 2018, a series of Lancet publications defined LBP more as a symptom than a disease that could be associated with a wide range of underlying disease conditions or physical abnormalities. LBP is characterized by pain between the lower rib edge and gluteal folds, often extending to lower limbs (2). Etiologically, LBP can be categorized into specific and non-specific types (3). Specific LBP has identifiable pathological causes such as tumors, infections, metabolic diseases, inflammatory arthritis, and fractures (4). While, non-specific LBP, accounting for 90% of cases, arises from unknown pathological or anatomical causes (3, 5).

LBP may involve several etiopathological factors, including intervertebral disc (IVD) degeneration, ligament overstretching, nerve root compression, and paraspinal muscle (PSM) degeneration (6). IVD degeneration, a primary cause of LBP symptoms, affects approximately 26%–42% of patients (7). IVD degeneration is characterized by an imbalance between synthesis and degradation processes in the IVD. The pathological hallmarks of IVD degeneration include alterations in extracellular matrix components, loss of proteoglycans and water content in the nucleus pulposus, protrusions of the nucleus pulposus, tears in the annulus fibrosus, and a subsequent reduction in the disc height (8, 9).

Furthermore, PSM pathology or degeneration can lead to LBP symptom onset (10). PSM, such as psoas, multifidus, and erector spinae, are vital for spinal stability and mobility (11). The multifidus muscle, which extends nearly the entire length of the spine, is especially susceptible to wear and degeneration (9). The quality and integrity of PSM are generally assessed by measuring the muscle cross-sectional area (CSA) and fat infiltration (FI) rate, where CSA indicates the muscle size and FI reveals the extent of fat deposition in muscles, serving as the key determining factor for PSM degeneration development (12). Importantly, Urrutia et al. revealed a direct correlation between PMS-FI and the extent of IVD degeneration (13).

Compared to healthy individuals, non-specific LBP patients often exhibit significant tissue-level differences in multifidus muscle structures, including variations in muscle fiber type compositions, fiber diameter, and CSA (14). Adult mammalian skeletal muscles typically consist of three primary fiber types, categorized by their functional and metabolic traits: type I (slow-twitch), type IIA (fast-twitch oxidative), and type IIX (formerly known as type IIB, fast-twitch glycolytic) (15). Mannion et al. demonstrate that the PSM is predominantly composed of type I fibers. For instance, in males, the thoracic PSM shows a distribution pattern of 62.0% type I, 26.8% type IIA, and 10.9% type IIX, and the lumbar region contains 65.0% type I, 24.2% type IIA, and 9.6% type IIX; while in females, thoracic PSM includes 67.8% type I, 27.3% type IIA, and 4.6% type IIX, and the lumbar region muscle comprises of 63.6% type I, 26.9% type IIA, and 9.0% type IIX fibers, reflecting optimal skeletal muscle fiber compositions for different genders and anatomical locations (15). whereas, LBP patients exhibit a marked increase in type IIX fibers and a concomitant reduction in type I fibers (15).

Therefore, more in-depth foundational studies are warranted to elucidate the relationship between multifidus and the onset of non-specific LBP. Due to ethical constraints, certain studies cannot be conducted on humans, necessitating the application of relevant animal models to mimic human disease progression and investigate potential therapeutic avenues. This approach has significantly advanced the field of animal model research (16). However, the underlying pathomechanism of IVD degeneration in the multifidus muscle, leading to LBP, remains unclear. Consequently, animal models are vital for further processes and exploring future therapies (17). Commonly used animal models for IVD degeneration include rodents, rabbits, dogs, sheep, pigs, and primates (18). Although most literature reviews mainly focused on analyzing structural changes in the multifidus muscle of LBP patients (14, 19), no systematic review has addressed the challenges in selecting appropriate animal models of IVD degeneration. Therefore, we conducted this systematic review of published research studies involving animal models to assess macroscopic and microscopic structural changes in the multifidus muscle in the context of IVD degeneration, aiming to establish an improved and standardized method for IVD degeneration model construction and evaluation. This study lays the foundation for further investigation of the pathological implication of PSM degeneration in subsequent induction of IVD degeneration.

## 2 Materials and methods

### 2.1 Methodology

This systematic review was conducted by strictly adhering to the Preferred Reporting Items for Systematic Reviews and Meta-Analyses (PRISMA) guidelines, as described in Supplementary Appendix 1 (20). Given the nature of this systematic review of published research articles, this study was waived for the requirement of ethical approval.

### 2.2 Eligibility criteria

#### 2.2.1 Inclusion criteria

Research studies were included for analysis only if they met the following criteria: (1) focused on animal models of IVD degeneration, without any species, age, or gender restrictions; (2) provided a detailed description of experimental methods highlighting the impact of IVD degeneration on secondary multifidus degeneration, especially structural changes, in a diseased animal model; (3) reported at least one of the following outcomes: mass of multifidus muscle, FI, stiffness, fiber type composition, and fiber CSA; and (4) published in the English language in a peer-reviewed journal indexed in the concerned databases.

#### 2.2.2 Exclusion criteria

Studies meeting any of the following criteria were excluded: (1) studies without original data; (2) studies lacking either an IVD degeneration animal model or documentation of structural changes in the multifidus muscle; (3) studies with an inadequate, unclear, or insufficient description of methods; and (4) duplicate or very similar studies.

### 2.3 Information sources

After establishing the inclusion and exclusion criteria for this study, two researchers conducted a comprehensive systematic search across several electronic medical databases, including PubMed, EMBASE, Web of Science (WoS), Cochrane Library, and MEDLINE Ovid. Literature searches were continued through November 2023, with articles systematically screened based on titles, abstracts, and full texts. Additionally, manual searches of the reference lists of identified studies ensured that no relevant research studies were overlooked.

### 2.4 Search strategy

The search strategy for this study was developed based on PubMed guidelines. To minimize the risk of omitting relevant literature, we employed “MeSH” terms to define specific search topics. We used Boolean operators AND and OR to combine MeSH terms with other search terms, refining the scope of the search and enhancing the precision of the results. This strategy was adapted to the specific requirements of the other four databases, as detailed in Supplementary Appendix 2.

### 2.5 Selection process

The study selection process was conducted independently by two researchers, strictly adhering to the inclusion and exclusion criteria. Full-length research articles were progressively screened based on the title, abstract, and full text, followed by cross-checking the results after the initial screening phase. Subsequent refinements and verifications were performed by the third and fourth reviewers. In cases of disagreement, the researchers first discussed the discrepancy to reach a consensus. If the matter was not resolved, an tenth researcher was introduced to the team to make the final decision.

### 2.6 Data items and extraction

After completing the full-text screening, data extraction was independently carried out by two researchers from the selected studies. The extracted data were then compared and verified for integrity and accuracy by two additional researchers. Any discrepancies were resolved through discussions or consultation with a senior researcher. Collected data included essential details such as the first author, affiliations, publication year, study title, and research objectives, in addition to specifics about the study animals, including species, gender, age, and body weight, as well as general study characteristics, sample size, modeling methods, control groups, observation period, vertebral level, and assessment methods. The primary outcomes were muscle mass, CSA, FI, stiffness, fiber type composition, and fiber CSA. A comprehensive summary of these details is available in Supplementary Table 1.

### 2.7 Risk of bias assessment

Two researchers rigorously evaluated the quality and risk of bias of selected studies using the Systematic Review Centre for Laboratory Animal Experimentation's Risk of Bias (SYRCLE's RoB) tool (21), which assessed six domains, namely selection bias, performance bias, detection bias, attrition bias, reporting bias, and other biases. These domains were then divided into ten items and supplemented with a list of additional signaling questions to facilitate the assessment. The ratings of “yes,” “no,” or “unclear” answers corresponded to low, high, and unclear biases, respectively, where a higher number of “yes” responses indicated a higher study quality. Any discrepancies during the evaluation were resolved by a senior researcher. The assessments were performed using Review Manager (RevMan) version 5.4 software. Detailed results can be found in Supplementary Figure 1.

## 3 Results

### 3.1 Study selection

After conducting an extensive systematic search across multiple electronic medical databases, we initially identified 89 relevant studies from PubMed (*n* = 14), EMBASE (*n* = 16), WoS (*n* = 46), Cochrane Library (*n* = 0), and MEDLINE Ovid (*n* = 13). Another study was identified through citation searching and included in the screening process. After removing 34 duplicate articles, 56 studies were screened for title and abstract relevance. Then, 20 studies were screened out for the full-text analysis. Next, 11 articles were excluded based on inclusion and exclusion criteria as decided by two researchers. Finally, nine studies met the PRISMA standards and were included in the systematic review (22–30). The complete screening process and specific reasons for exclusions/inclusions at each stage are illustrated in Supplementary Figure 2.

### 3.2 Study characteristics

The studies selected for the final analysis encompassed a publication span of 35 years, from 1988 (22) to 2022 (23, 29). Notably, two-thirds of those articles (6 out of 9) shared a common author, Paul W. Hodges (24, 25, 27–30) Detailed characteristics of the included studies are summarized in Supplementary Table 1.

#### 3.2.1 Animal models and modeling methods

This analysis included nine studies that categorized animal models of IVD degeneration by species. Notably, mice and rats were the most common species used in four studies (44.45%) (22, 23, 25, 28). The second most prevalent model was the castrated Merino sheep, utilized in three studies (33.33%) (24, 27, 29). The remaining two studies employed New Zealand white rabbits (26) or domestic pigs (30) as models in each study (11.11%). Moreover, all nine studies comprehensively detailed the methodologies for constructing IVD degeneration rodent models. The most frequently used method was the surgical blade injury (55.56%) (24, 25, 27, 29, 30), followed by needle puncture (33.33%) (23, 26, 28). One study combined needle puncture with a spontaneous degeneration model (28). Another distinct approach was the use of upper limb amputation establishing a bipedal walking rat model (22). Detailed results of the studies are presented in Supplementary Table 1.

#### 3.2.2 Observation time and vertebral level

The observation periods in nine studies varied from three days (30) to 18 months (22) post-injury, based on the species type. Notably, four studies (44.44%) (23, 24, 27, 29) commonly selected the six months post-injury observation time point. For post-operative specimen collection and radiological evaluations, rodents were examined from L3 to S1 (22, 23, 25, 28), with L4 and L5 being the most frequently assessed segments in all four rodent studies; castrated Merino sheep were evaluated for L1-2, L3-4, and L5-6 segments in three studies (24, 27, 29); New Zealand white rabbits were assessed for L2-3 and L4-5 (26), and domestic pigs for L3-4 (30) segmental degeneration pathology. It is noteworthy that segments L4 and L5 were the most commonly evaluated regions across various species. Detailed results of the studies are presented in Supplementary Table 1.

#### 3.2.3 Macroscopic changes

This study encompasses nine research articles that macroscopically assessed the multifidus muscle using various parameters: muscle mass in one study (11.11%) (25), CSA in two studies (22.22%) (27, 30), FI in four studies (44.44%) (23, 26, 27, 30), and stiffness in one study (11.11%) (26). The detailed evaluation results are described below.

##### 3.2.3.1 Muscle mass

Of the nine included studies, only one investigated multifidus muscle mass. Maas et al. developed an adult male Wistar rat model of IVD degeneration by surgically inducing damage at the L4/L5 segments. Then, bilateral multifidus muscle samples were collected from L3/L4, L4/L5, and L5/L6 segments at 7, 14, and 28 days post-injury to measure their mass. The findings revealed that the multifidus muscle mass in the IVD degeneration group was significantly reduced by 20% compared to the control group, in seven days post-surgery (*p* < 0.05) (25). However, the difference was not statistically significant between the groups on days 14 and 28 post-surgery (*p* > 0.05) (25). Detailed results of the studies are presented in Supplementary Table 1.

##### 3.2.3.2 CSA

Two of the nine studies assessed the CSA of multifidus muscles for any potential muscle atrophy. Hodges et al. conducted these studies first using a domestic pig model (30) and later with a castrated Merino sheep model (27). Both studies measured bilateral CSA through ultrasound (30) and histological techniques (27) to distinguish between damaged and non-damaged muscle sides in respective animal models. However, those studies revealed inconsistent findings such that the 2006 study reported a significant (17%) reduction in CSA at the L4 spinous process on the damaged side three days post L3-L4 IVD injury (*p* < 0.001) (30), whereas the 2015 study found no significant changes in CSA six months post-injury following left-sided IVD surgery at levels L1-2, L3-4, and L5-6. Furthermore, histological analysis confirmed the absence of any insignificant increases in CSA (*p* = 0.315) (27). Detailed results of the studies are presented in Supplementary Table 1.

##### 3.2.3.3 FI

Four studies investigated the extent of FI of the multifidus muscle using histological methods (23, 26, 27, 30). One study also employed the quantitative magnetic resonance imaging (qMRI) technique to scan the multifidus muscle (23) and analyzed the data using vendor-supplied algorithms to determine the proton density fat fraction (PDFF). These studies uniformly concluded that there was an increase in intramuscular gaps, enhanced accumulation of intermuscular fat, a larger CSA of fat tissue, and more severe FI in the multifidus muscle. Detailed results of the studies are presented in Supplementary Table 1.

##### 3.2.3.4 Stiffness

One of the nine studies reviewed assessed the stiffness of the multifidus muscle (26). Brown et al. performed passive mechanical tests on the multifidus muscle at the L3 and L7 levels of New Zealand white rabbits, revealing a significant increase in stiffness at these levels. Of note, a higher stiffness was observed in the 12-week IVD degeneration group compared to both the 4-week IVD degeneration and control groups (*p* < 0.0001). Detailed results of the studies are presented in Supplementary Table 1.

#### 3.2.4 Microscopic changes

Out of nine studies, six studies assessed microstructural alterations in the multifidus muscle fibers in terms of fiber type compositions (66.67%) (22, 24, 26–29) and diameter or CSA (33.33%) (27–29) The specific results of these evaluations are presented below:

##### 3.2.4.1 Fiber type composition

Six of the nine studies assessed the fiber type composition of the multifidus muscle (22, 24, 26–29)—two studies employed histopathological (22, 29) and the other four applied immunohistochemical (IHC) techniques (24, 26–28) Three studies found that IVD degeneration animals had a significantly decreased proportion of type I fibers, whereas type II fibers exhibited a significant increase (22, 24, 28). Notably, the other three studies presented contradictory findings, observing no statistically significant changes in muscle fiber compositions at specified post-modeling time points. Particularly, Brown et al. reported no significant differences in type I fiber percentages between the control and 12-week IVD degeneration groups but not between the L3 and L7 segments (26). Hodges et al. also demonstrated no significant changes in fiber type ratios in three months post-modeling (all *p* > 0.153) (27). Similarly, James et al. could not detect any significant differences in the proportions of the nuclear bag (*p* = 0.25) and chain fibers (*p* = 0.31) between the control and IVD degeneration groups (29). Detailed results of the studies are presented in Supplementary Table 1.

##### 3.2.4.2 Fiber size or CSA

Among the three studies examining the diameter or CSA of multifidus muscle fibers, two conducted IHC (27, 28) and one employed histological methods (29). Interestingly, the experimental outcomes of these studies led to different conclusions. Hodges et al. found no statistically significant differences in CSA of type I fibers between the control and IVD degeneration groups at three (all *p* > 0.114) and six months (all *p* > 0.605) post-injury (27). In contrast, James et al. reported a significantly lower CSA of type I fibers in the IVD degeneration group compared to the control group (*p* < 0.005) (28). However, the same author observed no significant changes in the CSA of nuclear bag fibers (*p* = 0.45) between the groups in a later study, although the CSA of nuclear chain fibers was significantly larger in the IVD degeneration group (*p* = 0.020) (29). Detailed results of the studies are presented in Supplementary Table 1.

### 3.3 Risk of bias

Two researchers independently assessed the quality of nine animal model-related studies using the SYRCLE's RoB tool, which revealed a lower risk of bias in 36.67%, an unclear risk in 58.89%, and a higher risk in 4.44% of cases. Concerning the selection bias, all studies failed to adequately describe random sequence generation and allocation concealment, thereby receiving an “unclear risk of bias” rating. Of these, three studies (33.33%) (24, 27, 29) reported balanced baseline characteristics between the control and experimental groups, meriting a “low risk of bias.” In the area of performance bias, four studies (44.44%) (23–25, 27) ensured that experimental animals were housed under identical conditions, aligning with randomization principles and thus were also rated as “low risk of bias.” However, three studies (33.33%) (26, 29, 30) did not disclose the living conditions of the experimental animals, resulting in an “unclear risk of bias”, and two studies (22.22%) (22, 28) reporting their study animal housing in different environments were designated with a “high risk of bias.” Furthermore, the majority (88.89%) (23–30) of studies did not specify whether blinding was implemented for animal caregivers and researchers, thus resulting in an “unclear risk of bias” rating. Only one study (11.11%) (22) explicitly mentioned the inability to blind personnel due to the special design of cages and was marked as “high risk of bias”. Regarding the detection bias, none of these studies detailed the implementation of proper randomization methods for selecting animals for outcome assessment, leading to an “unclear risk of bias.” Additionally, four studies (44.44%) (22, 26, 29, 30) which blinded their outcome assessors, were classified as “low risk of bias,” while the remaining five (55.56%) studies (23–25, 27, 28) did not clarify this point, thus receiving an “unclear risk of bias” recognition. Regarding the attrition bias evaluation, one study (11.11%) (28) reported having an incomplete dataset due to unexpected death or exclusion of animals without addressing the impact on study integrity, earning a “high risk of bias.” Four studies (22, 25, 27, 30) reported complete outcome data and were thus assessed as “low risk of bias,” whereas the other four (23, 24, 26, 29) lacked explicit reporting on data completeness, resulting in an “unclear risk of bias.” All studies were evaluated as “low risk” for reporting and other biases. Detailed results of the bias risk assessments are available in Supplementary Figure 1.

## 4 Discussion

Globally, LBP is a common clinical symptom and ranks among the top ten reasons for visiting emergency departments, including in the United States and Canada. LBP can significantly diminish the patient's work capacity and quality of life (QoL) while consuming substantial healthcare resources. Current statistical analyses indicate a lifetime prevalence of 39.9% ± 24.3% of LBP, with higher incidence rates in elderly females (40–80 years) (31–34). Despite incomplete understanding of its pathogenesis, IVD and PMS degeneration are recognized as crucial contributors. Recently, a study investigated the relationship between PSM quality and LBP (9), highlighting PMS's role in spinal stability and function. Deterioration in PMS, characterized by reduced CSA, increased FI, and fiber type transformation, exacerbates LBP. Previous studies have examined the causal relationship between IVD and PMS degeneration using animal models. For example, Maas et al. (2018) found a significant reduction in multifidus muscle weight by postoperative day 7 in the IVD degeneration group compared to the control (*p* < 0.05) group, while no significant IVD degeneration was detected in the multifidus excision group. The authors suggest that the time course and self-recovery rate of rat IVD might account for varying observations, thereby hypothesizing a causal relationship between IVD degeneration onset and PSM degeneration induction. Nevertheless, additional experiments are necessary to confirm this relationship (25). Furthermore, PSM denervation, which can occur asymptomatically, may be morphologically susceptible to neural stretching after disc height loss, leading to denervation and subsequent PSM degeneration. This neuro-mechanical basis for the decline in muscle quality and the progression of IVD degeneration highlights the interconnected nature of these degenerative processes.

This study presents a systematic review of existing experimental studies on animal models of IVD degeneration, specifically examining the structural changes in the multifidus after IVD degeneration development. We critically assessed various factors, including the selection of model animals, modeling methods, and evaluation criteria for multifidus structural changes. The objective of this study was to propose enhanced methodologies for developing IVD degeneration models and evaluating standards for multifidus degeneration in future research. This review aims to explore the causal link between IVD and PSM degeneration, clarify the pathophysiological mechanisms of LBP, and potentially identify novel clinical approaches for effective LBP management.

### 4.1 Species selection for animal models

The selection of appropriate animal models is pivotal for effective IVD degeneration research. Commonly, rats, mice, guinea pigs, hamsters, rabbits, dogs, cattle, sheep, goats, domestic pigs, chickens, horses, camels, kangaroos, and primates, such as baboons and macaques are exploited to develop the disease model (18, 35, 36). However, the majority of these models are quadrupeds, and their habitual postures and locomotive behaviors, influenced by gravity, significantly differ from those of humans. Even bipeds, such as primates and kangaroos, exhibit variations in posture and movement from humans; for example, kangaroos leverage their tails for spinal support, potentially mitigating IVD and PSM degeneration. Therefore, these animal models cannot fully replicate the natural degenerative processes observed in human IVD degeneration pathology.

In 1929, Colton et al. recognized the potential of bipedal models and developed a method to induce bipedalism in rats by forelimb amputation (37). Since then, this approach has gained traction, with numerous researchers refining the model to significantly enhance IVD degeneration outcomes (38–40). For instance, Liang et al. established a similar model using brachial plexus root avulsion (41, 42), while Ao et al. induced a bipedal posture in mice by leveraging their aversion to water, placing them in containers with a water depth of 5mm (43). While, Cassidy et al. opted for a bipedal rat model with ligation and excision of upper limbs above the humerus, and coupled with housing in specially designed cages that required standing for access to elevated food and water sources (22). By six weeks of age, these rats were capable of walking entirely upright and spent considerable time each day in this posture, thus closely mimicking the natural progression of IVD degeneration observed in humans.

### 4.2 Modeling methods

Various methods have been developed by several research groups to model IVD degeneration animals beyond the bipedal rats, as described previously. These include the needle puncture method to injure the IVD; the blade injury model using surgical blades to disrupt the IVD structure; and the spinal instability model, which involves separating the posterior muscle complex, excising spinous processes, and severing ligaments. Other techniques include the tail curling/ring model, inducing force imbalance in IVD; the axial compression model, applying pressure under compressed and angled conditions; and the vibration model, subjecting animals to continuous vibrational forces. Furthermore, the injection of specific chemicals can also induce IVD degeneration and a combined application of aforementioned methods can also be exploited to establish an effective IVD degeneration model in rodents. Genetic modifications, such as in SPARC-null mice, SM/J mice, and LG/J mice, can disrupt IVD metabolism and induce loss of structural integrity, closely simulating natural degeneration processes in humans. Besides, the aging model of IVD degeneration focuses on the age-related structural and pathological changes in hamsters (18, 35, 36, 44–58).

This study aimed to underscore the importance of structural changes in the multifidus muscle in animal models of IVD degeneration. Of note, it is critical to minimize any impact on the multifidus muscle during the model construction. Consequently, the posterior approach is inappropriate as it inevitably damages the PSM, at least to some extent. On the other hand, the method analyzed in this study involved the slow insertion of needles through the rat's back into the L4/5 and L5/6 IVD segments. For the sham group, needles were inserted to pierce only the PSM without penetrating the IVD structure, thereby isolating the effect of PSM puncture itself (23). This technique simulates common clinical manifestations of human posterior lateral IVD lesions. Nonetheless, in most cases, artificial disturbances in PSM could be a major confounding factor leading to PSM degeneration secondary to the IVD degeneration.

Although the bipedal rat model closely resembles the natural degenerative processes of human IVD degeneration, however, is limited to its widespread application due to ethical concerns, modeling challenges, and prolonged observation periods. Recently, genetically modified and aging models have become the most effective disease animal models with spontaneous IVD degeneration without requiring any external physical or chemical interventions (28). However, the multifactorial nature of IVD degeneration pathology and the non-specific triggering of unknown pathophysiological mechanisms are the major bottlenecks in the widespread application of gene knockout IVD degeneration models (44). In contrast, anterior approach needle puncture and blade injury methods are globally recognized for IVD degeneration modeling due to their straightforward techniques, significant IVD degeneration post-modeling, and minimal interference with PMS damage.

### 4.3 Vertebral level selection

When selecting the segmental level of IVD for model construction, it is important to consider the physio-anatomical structure of the modeling animal due to significant interspecies variations. In humans, degeneration of lumbar IVD predominantly occurs at the L4-L5 and L5-S1 levels, where 90% of disc herniations manifest, largely because of the sacro-lumbar intermediate position. Unlike lumbar vertebrae, sacral vertebrae in adults typically fuse into a single sacrum, lacking physiological movement and buffering capabilities. Therefore, subjecting the L4-L5 and L5-S1 IVDs to increased mechanical pressures and motion ranges, particularly during flexion and extension, can promote the IVD degeneration onset (59–62). Thus, it is advisable to select the two most distal segments of the lumbar spine to model IVD degeneration in animals. Experimental studies cited in this article, informed by relevant zoological literature, note that rodents usually possess 6 lumbar vertebrae, whereas rabbits have 7, and sheep and domestic pigs 6–7 (63–71). In all the nine studies reviewed, segments near the distal lumbar spine, especially L4 and L5, were consistently selected.

### 4.4 Assessment systems

The severity of both LBP and IVD degeneration is closely correlated with the quality of the PSM (72). Consequently, establishing an effective evaluation system for PSM degeneration is crucial for numerous animal models of IVD degeneration to accurately assess the model's effectiveness. The PSM, comprising critical muscle groups surrounding the spine, including the multifidus, psoas, and erector spinae, plays a vital role in maintaining spinal stability and supporting physiological movement. A decline in PSM function can disrupt biomechanical balance and structural stability of the spine, leading to uneven stress on the IVD and accelerating its degenerative processes (73–76) PSM degeneration primarily manifests as reduced muscle mass and CSA, increased FI and stiffness, decreased muscle fiber diameter, changes in muscle fiber type compositions, and alterations in various inflammatory cytokines and gene expression levels. Patients with PSM degeneration exhibit decreased muscle strength, endurance, and plasticity (77–81). Among the nine studies analyzed, five studies evaluated the multifidus muscle from a macroscopic perspective. Overall, the study results support our initial hypothesis that at specific time points, IVD degeneration animals would exhibit significant levels of atrophy of the multifidus muscle, with marked reductions in mass and CSA, compared to sham animals. MRI and IHC revealed significant enlargement of multifidus muscle gaps and increased intramuscular lipid droplets in these animals. Furthermore, mechanical passive dynamic testing showed a significant increase (107%) in the stiffness of the multifidus in damaged IVD segments and their adjacent segments, compared to individual fibers (34% increase compared to control) (23, 25–27, 30). However, contrary to the expected hypothesis, Hodges et al. could not detect any significant atrophy of the multifidus at three and six months post-operative injury to the left IVD of Merino sheep via a retroperitoneal approach. The authors suggest that the influence of pro-inflammatory cytokines might prevent muscle atrophy during the transition from the subacute (3 months) to early chronic stages (6 months) of IVD injury (27). Some studies indicate that FI is more significantly associated with high-intensity pain, functional impairments, and abnormalities in spinal structure than muscle CSA, thus potentially serving as an effective indicator for evaluating muscle degeneration (12, 13, 82–84).

At the microscopic level, muscle quality assessment predominantly focuses on the CSA of muscle fibers and their type of distribution. Skeletal muscle fibers are categorized into two major subtypes: type I (slow-twitch) fibers, which primarily utilize aerobic metabolism for energy production, and exhibit higher endurance levels appropriate for prolonged physical activities such as posture maintenance; and type II (fast-twitch) fibers rely on anaerobic metabolism for rapid energy generation and are essential for activities that require short bursts of power like jumping or sprinting (85–87). In bipedal upright species such as humans and primates, lumbar muscles, bearing significant weight, predominantly consist of type I fibers. In contrast, quadrupeds like rodents, dogs, sheep, and horses mainly possess type II fibers (88–92). Research shows that the proportion of type I fibers in human multifidus muscles ranges from 54% to 63% (24, 80, 93), whereas in sheep, it is only 16% to 28% (24, 27, 29), and in rodents, it drops to 5% to 15% (28, 94). Experiments by Cassidy et al. on bipedal upright rats demonstrated a significant increase in type I fibers in their multifidus muscles, while muscle biopsies of human LBP patients indicate a lower proportion of type I fibers and thus poorer fatigue resistance, negatively correlating with the duration of pain (28, 95, 96). Recent research has expanded to include gene expressions of cytokines such as TNF-α and IL-6 to explore the specific pathophysiological mechanisms of LBP and the potential links between IVD and multifidus degeneration (23, 24, 27, 97, 98). Moreover, several studies incorporate behavioral assessments, including gait analysis, von Frey pain tests, hot plate tests, radiant heat tests, acetone tests, spontaneous pain tests, grip tests, and tail suspension tests, to comprehensively evaluate pain symptoms and muscle dysfunction in animal models (23, 99–104).

In summary, we propose the inclusion of several criteria in future evaluations of PSM degeneration: Firstly, radiologically, the assessment of CSA and FI are essential parameters. Secondly, the transition of muscle fiber types and their proportionate composition should be evaluated using immunohistochemical staining. Additionally, a behavioral assessment of muscle degeneration is warranted, encompassing strength, coordination, and motor function, as measured by the Basso-Beattie-Bresnahan Locomotor Rating Scale, grip strength test, hot plate test, and tail suspension test, to fully understand the impact on animal behavior. Finally, post-mortem, changes in inflammatory cytokines and gene expression levels, including TNF-α, IL-6, IL-1β, miRNA, and TGF-β, should be examined to assess muscle degeneration. We posit that these multidimensional indicators can form a scientifically robust evaluation system, offering a reference and theoretical foundation for research into IVD and PSM degeneration models.

## 5 Conclusion

This systematic review offers a detailed analysis of structural changes in the multifidus muscle across various animal models of IVD degeneration. Further, this study outlines the criteria for the selection of model animals, methods of modeling, and evaluation battery, providing essential insights into the advancement of IVD degeneration model development and assessment methods for multifidus muscle degeneration. Despite the limited number of studies and the inherent heterogeneity, that prevented a quantitative meta-analysis, this review addresses current research limitations and outlines future research directions. Notably, a significant gap remains in understanding the biomechanical properties and pathophysiological mechanisms of multifidus muscle degeneration. Future research should concentrate on these areas to enhance our understanding of the potential interactions between IVD degeneration and multifidus muscle dysfunction, clarifying the pathophysiological mechanisms behind LBP, and fully elucidating the pathological processes of spinal degenerative diseases. Such efforts will underpin the development of novel therapeutic strategies.

## Data Availability

The original contributions presented in the study are included in the article/Supplementary Material, further inquiries can be directed to the corresponding author.

## References

[1] GBD 2021 Low Back Pain Collaborators. Global, regional, and national burden of low back pain, 1990–2020, its attributable risk factors, and projections to 2050: a systematic analysis of the global burden of disease study 2021. Lancet Rheumatol. (2023) 5:e316–29. 10.1016/S2665-9913(23)00098-X 37273833 PMC10234592

[2] Hartvigsen J Hancock MJ Kongsted A Louw Q Ferreira ML Genevay S What low back pain is and why we need to pay attention. Lancet (London, England). (2018) 391(10137):2356–67. 10.1016/S0140-6736(18)30480-X 29573870

[3] Koes BW van Tulder MW Thomas S. Diagnosis and treatment of low back pain. Br Med J. (2006) 332:1430–4. 10.1136/bmj.332.7555.1430 16777886 PMC1479671

[4] Kreiner DS Matz P Bono CM Cho CH Easa JE Ghiselli G Guideline summary review: an evidence-based clinical guideline for the diagnosis and treatment of low back pain. Spine J. (2020) 20(7):998–1024. 10.1016/j.spinee.2020.04.006 32333996

[5] Maher C Underwood M Buchbinder R. Non-specific low back pain. Lancet. (2017) 389:736–47. 10.1016/S0140-6736(16)30970-9 27745712

[6] Alleva J Hudgins T Belous J Kristin Origenes A. Chronic low back pain. Dis Mon. (2016) 62:330–3. 10.1016/j.disamonth.2016.05.012 27344193

[7] Isa M Teoh IL Mohd Nor SL & Mokhtar NH A S. Discogenic low back pain: anatomy, pathophysiology and treatments of intervertebral disc degeneration. Int J Mol Sci. (2023) 24:208. 10.3390/ijms24010208 PMC9820240 36613651

[8] Krut Z Pelled G Gazit D Gazit Z. Stem cells and exosomes: new therapies for intervertebral disc degeneration. Cells. (2021) 10:2241. 10.3390/cells10092241 34571890 PMC8471333

[9] Wang Z Zhao Z Han S Hu X Ye L Li Y Advances in research on fat infiltration and lumbar intervertebral disc degeneration. Front Endocrinol (Lausanne). (2022) 13:1067373. 10.3389/fendo.2022.1067373 36568091 PMC9768030

[10] Wesselink EO Pool JJM Mollema J Weber KA 2nd Elliott JM Coppieters MW Is fatty infiltration in paraspinal muscles reversible with exercise in people with low back pain? A systematic review. Eur Spine J. (2023) 32(3):787–96. 10.1007/s00586-022-07471-w 36459201 PMC10515728

[11] Hodges PW Danneels L. Changes in structure and function of the back muscles in low back pain: different time points, observations, and mechanisms. J Orthop Sports Phys Ther. (2019) 49(6):464–76. 10.2519/jospt.2019.8827 31151377

[12] Suo M Zhang J Sun T Wang J Liu X Huang H The association between morphological characteristics of paraspinal muscle and spinal disorders. Ann Med. (2023) 55(2):2258922. 10.1080/07853890.2023.2258922 37722876 PMC10512810

[13] Urrutia J Besa P Lobos D Campos M Arrieta C Andia M Lumbar paraspinal muscle fat infiltration is independently associated with sex, age, and inter-vertebral disc degeneration in symptomatic patients. Skeletal Radiol. (2018) 47(7):955–61. 10.1007/s00256-018-2880-1 29379999

[14] Stevens S Agten A Timmermans A Vandenabeele F. Unilateral changes of the multifidus in persons with lumbar disc herniation: a systematic review and meta-analysis. Spine J. (2020) 20:1573–85. 10.1016/j.spinee.2020.04.007 32325246

[15] Mannion AF. Fibre type characteristics and function of the human paraspinal muscles: normal values and changes in association with low back pain. J Electromyogr Kinesiol. (1999) 9:363–77. 10.1016/S1050-6411(99)00010-3 10597049

[16] Mukherjee P Roy S Ghosh D Nandi SK. Role of animal models in biomedical research: a review. Lab Anim Res. (2022) 38(18):18. 10.1186/s42826-022-00128-1 35778730 PMC9247923

[17] Daly C Ghosh P Jenkin G Oehme D Goldschlager T. A review of animal models of intervertebral disc degeneration: pathophysiology, regeneration, and translation to the clinic. Biomed Res Int. (2016) 2016:5952165. 10.1155/2016/5952165 27314030 PMC4893450

[18] Poletto DL Crowley JD Tanglay O Walsh WR Pelletier MH. Preclinical *in vivo* animal models of intervertebral disc degeneration. Part 1: a systematic review. JOR Spine. (2023) 6:e1234. 10.1002/jsp2.1234 36994459 PMC10041387

[19] Danneels L. Structural changes of lumbar muscles inNon-specific low back pain. Pain Phys. (2016) 7;19:E985–E1000. 10.36076/ppj/2016.19.E985 27676689

[20] Page MJ McKenzie JE Bossuyt PM Boutron I Hoffmann TC Mulrow CD The PRISMA 2020 statement: an updated guideline for reporting systematic reviews. BMJ (Clin Res Ed). (2021) 372:n71. 10.1136/bmj.n71 PMC8005924 33782057

[21] Hooijmans CR Rovers MM de Vries RB Leenaars M Ritskes-Hoitinga M Langendam MW. SYRCLE’s risk of bias tool for animal studies. BMC Med Res Methodol. (2014) 14:43. 10.1186/1471-2288-14-43 24667063 PMC4230647

[22] Cassidy JD Yong-Hing K Kirkaldy-Willis WH Wilkinson AA. 1. A study of the effects of bipedism and upright posture on the lumbosacral spine and paravertebral muscles of the wistar rat. SPINE. (1988) 13:301–8. 10.1097/00007632-198803000-00013 3388116

[23] Huang Y Wang L Luo B Yang K Zeng X Chen J Associations of lumber disc degeneration with paraspinal muscles myosteatosis in discogenic low back pain. Front Endocrinol (Lausanne). (2022) 13:891088. 10.3389/fendo.2022.891088 35634490 PMC9136003

[24] Hodges PW James G Blomster L Hall L Schmid AB Shu C Can proinflammatory cytokine gene expression explain multifidus muscle fiber changes after an intervertebral disc lesion? Spine. (2014) 39(13):1010–7. 10.1097/BRS.0000000000000318 24718080

[25] Maas H Noort W Hodges PW van Dieen J. Effects of intervertebral disc lesion and multifidus muscle resection on the structure of the lumbar intervertebral discs and paraspinal musculature of the rat. J Biomech. (2018) 70:228–34. 10.1016/j.jbiomech.2018.01.004 29395230

[26] Brown SH Gregory DE Carr JA Ward SR Masuda K Lieber RL. ISSLS Prize winner: adaptations to the multifidus muscle in response to experimentally induced intervertebral disc degeneration. Spine. (2011) 36(21):1728–36. 10.1097/BRS.0b013e318212b44b 21301396

[27] Hodges PW James G Blomster L Hall L Schmid A Shu C Multifidus muscle changes after back injury are characterized by structural remodeling of muscle, adipose and connective tissue, but not muscle atrophy: molecular and morphological evidence. Spine. (2015) 40(14):1057–71. 10.1097/BRS.0000000000000972 25943090

[28] James G Millecamps M Stone LS Hodges PW. Multifidus muscle fiber type distribution is changed in mouse models of chronic intervertebral disc degeneration, but is not attenuated by whole body physical activity. Spine. (2021) 46:1612–20. 10.1097/BRS.0000000000004105 33973565

[29] James G Stecco C Blomster L Hall L Schmid AB Shu CC Muscle spindles of the multifidus muscle undergo structural change after intervertebral disc degeneration. Eur Spine J. (2022) 31(7):1879–88. 10.1007/s00586-022-07235-6 35618974 PMC7613463

[30] Hodges P Holm AK Hansson T Holm S. Rapid atrophy of the lumbar multifidus follows experimental disc or nerve root injury. Spine. (2006) 31:2926–33. 10.1097/01.brs.0000248453.51165.0b 17139223

[31] Melman A Lord HJ Coombs D Zadro J Maher CG Machado GC. Global prevalence of hospital admissions for low back pain: a systematic review with meta-analysis. BMJ open. (2023) 13(4):e069517. 10.1136/bmjopen-2022-069517 37085316 PMC10124269

[32] Blyth FM Briggs AM Schneider CH Hoy DG March LM. The global burden of musculoskeletal pain—where to from here? Am J Public Health. (2019) 109:35–40. 10.2105/AJPH.2018.304747 30495997 PMC6301413

[33] Manchikanti L Singh V Falco FJE Benyamin RM Hirsch JA. Epidemiology of low back pain in adults. Neuromodulation. (2014) 17:3–10. 10.1111/ner.12018 25395111

[34] Hoy D Bain C Williams G March L Brooks P Blyth F A systematic review of the global prevalence of low back pain. Arthritis Rheum. (2012) 64(6):2028–37. 10.1002/art.34347 22231424

[35] Alini M Diwan AD Erwin WM Little CB Melrose J. An update on animal models of intervertebral disc degeneration and low back pain: exploring the potential of artificial intelligence to improve research analysis and development of prospective therapeutics. JOR Spine. (2023) 6:e1230. 10.1002/jsp2.1230 36994457 PMC10041392

[36] Wang Y Kang J Guo X Zhu D Liu M Yang L Intervertebral disc degeneration models for pathophysiology and regenerative therapy -benefits and limitations. J Invest Surg. (2022) 35(4):935–52. 10.1080/08941939.2021.1953640 34309468

[37] Colton HS. How bipedal habit affects the bones of the hind legs of the albino rat. J Exp Zool. (1929) 53:1–11. 10.1002/jez.1400530102

[38] Liu S Zhao B Shi H Liang Q Fu Y Yang Z Ligustrazine inhibits cartilage endplate hypertrophy via suppression of TGF-β1. Evid Based Complement Alternat Med. (2016) 2016:1042489. 10.1155/2016/1042489 27563332 PMC4985580

[39] Yao W Jee WS Chen J Liu H Tam CS Cui L Making rats rise to erect bipedal stance for feeding partially prevented orchidectomy-induced bone loss and added bone to intact rats. J Bone Miner Res. (2000) 15(6):1158–68. 10.1359/jbmr.2000.15.6.1158 10841185

[40] Kong M Zhang Y Song M Cong W Gao C Zhang J Myocardin-related transcription factor a nuclear translocation contributes to mechanical overload-induced nucleus pulposus fibrosis in rats with intervertebral disc degeneration. Int J Mol Med. (2021) 48(1):123. 10.3892/ijmm.2021.4956 33982787 PMC8121555

[41] Liang QQ Zhou Q Zhang M Hou W Cui XJ Li CG Prolonged upright posture induces degenerative changes in intervertebral discs in rat lumbar spine. Spine. (2008) 33(19):2052–8. 10.1097/BRS.0b013e318183f949 18758360

[42] Liang QQ Cui XJ Xi ZJ Bian Q Hou W Zhao YJ Prolonged upright posture induces degenerative changes in intervertebral discs of rat cervical spine. Spine. (2011) 36(1):E14–9. 10.1097/BRS.0b013e3181d2dec2 20948465

[43] Ao X Wang L Shao Y Chen X Zhang J Chu J Development and characterization of a novel bipedal standing mouse model of intervertebral disc and facet joint degeneration. Clin Orthop Relat Res. (2019) 477(6):1492–504. 10.1097/CORR.0000000000000712 31094848 PMC6554109

[44] Liang T Gao B Zhou J Qiu X Qiu J Chen T Constructing intervertebral disc degeneration animal model: a review of current models. Front Surg. (2023) 9:1089244. 10.3389/fsurg.2022.1089244 36969323 PMC10036602

[45] Rivera Tapia ED Meakin JR Holsgrove TP. *In vitro* models of disc degeneration – A review of methods and clinical relevance. J Biomech. (2022) 142:111260. 10.1016/j.jbiomech.2022.111260 36027637

[46] Masuda K Aota Y Muehleman C Imai Y Okuma M Thonar EJ A novel rabbit model of mild, reproducible disc degeneration by an anulus needle puncture: correlation between the degree of disc injury and radiological and histological appearances of disc degeneration. Spine. (2005) 30(1):5–14. 10.1097/01.brs.0000148152.04401.20 15626974

[47] Norcross JP Lester GE Weinhold P Dahners LE. An *in vivo* model of degenerative disc disease. J Orthop Res. (2003) 21:183–8. 10.1016/S0736-0266(02)00098-0 12507597

[48] Lotz JC Colliou OK Chin JR Duncan NA Liebenberg E. Compression-induced degeneration of the intervertebral disc: an *in vivo* mouse model and finite-element study. Spine (Phila Pa 1976). (1998) 23:2493–506. 10.1097/00007632-199812010-00004 9854748

[49] Oda H Matsuzaki H Tokuhashi Y Wakabayashi K Uematsu Y Iwahashi M. Degeneration of intervertebral discs due to smoking: experimental assessment in a rat-smoking model. J Orthop Sci. (2004) 9(2):135–41. 10.1007/s00776-003-0759-y 15045541

[50] Stokes IA Counts DF Frymoyer JW. Experimental instability in the rabbit lumbar spine. Spine (Phila Pa 1976). (1989) 14:68–72. 10.1097/00007632-198901000-00014 2913671

[51] Fukui D Kawakami M Yoshida M Nakao S Matsuoka T Yamada H. Gait abnormality due to spinal instability after lumbar facetectomy in the rat. Eur Spine J. (2015) 24(9):2085–94. 10.1007/s00586-014-3537-y 25186827

[52] Yurube T Nishida K Suzuki T Kaneyama S Zhang Z Kakutani K Matrix metalloproteinase (MMP)-3 gene up-regulation in a rat tail compression loading-induced disc degeneration model. J Orthop Res. (2010) 28(8):1026–32. 10.1002/jor.21116 20162718

[53] Sakai D Nishimura K Tanaka M Nakajima D Grad S Alini M Migration of bone marrow-derived cells for endogenous repair in a new tail-looping disc degeneration model in the mouse: a pilot study. Spine J. (2015) 15(6):1356–65. 10.1016/j.spinee.2013.07.491 25459743

[54] Sullivan JD Farfan HF Kahn DS. Pathologic changes with intervertebral joint rotational instability in the rabbit. Can J Surg. (1971) 14:71–9. 5540387

[55] Sahlman J Inkinen R Hirvonen T Lammi MJ Lammi PE Nieminen J Premature vertebral endplate ossification and mild disc degeneration in mice after inactivation of one allele belonging to the Col2a1 gene for type II collagen. Spine. (2001) 26(23):2558–65. 10.1097/00007632-200112010-00008 11725236

[56] McCann MR Patel P Pest MA Ratneswaran A Lalli G Beaucage KL Repeated exposure to high-frequency low-amplitude vibration induces degeneration of murine intervertebral discs and knee joints. Arthritis Rheumatol. (2015) 67(8):2164–75. 10.1002/art.39154 25891852

[57] Gruber HE Johnson T Norton HJ Hanley EN Jr. The sand rat model for disc degeneration: radiologic characterization of age-related changes: cross-sectional and prospective analyses. Spine. 27(3):230–4. 10.1097/00007632-200202010-00004 11805683

[58] Hadjipavlou AG Simmons JW Yang JP Bi LX Ansari GA Kaphalia BS Torsional injury resulting in disc degeneration: I. An *in vivo* rabbit model. J Spinal Disord. (1998) 11(4):312–7. 9726300

[59] Huang Y Wang L Zeng X Chen J Zhang Z Jiang Y Association of paraspinal muscle CSA and PDFF measurements with lumbar intervertebral disk degeneration in patients with chronic low back pain. Front Endocrinol (Lausanne). (2022) 13:792819. 10.3389/fendo.2022.792819 35721738 PMC9204273

[60] Wang Y Wang H Lv F Ma X Xia X Jiang J. Asymmetry between the superior and inferior endplates is a risk factor for lumbar disc degeneration. J Orthop Res. (2018) 36(9):2469–75. 10.1002/jor.23906 29611881

[61] Faur C Patrascu JM Haragus H Anglitoiu B. Correlation between multifidus fatty atrophy and lumbar disc degeneration in low back pain. BMC Musculoskelet Disord. (2019) 20:414. 10.1186/s12891-019-2786-7 31488112 PMC6729014

[62] Oichi T Taniguchi Y Oshima Y Tanaka S Saito T. Pathomechanism of intervertebral disc degeneration. JOR Spine. (2020) 3:e1076. 10.1002/jsp2.1076 32211588 PMC7084053

[63] Toossi A Bergin B Marefatallah M Parhizi B Tyreman N Everaert DG Comparative neuroanatomy of the lumbosacral spinal cord of the rat, cat, pig, monkey, and human. Sci Rep. (2021) 11(1):1955. 10.1038/s41598-021-81371-9 33479371 PMC7820487

[64] Skidanov A Ashukina N Maltseva V Skidanov M Danyshchuk Z Radchenko V. The relationship between structural changes in paraspinal muscles and intervertebral disc and facet joint degeneration in the lumbar spine of rats. J Orthop Surg Res. (2024) 19(1):58. 10.1186/s13018-024-04548-8 38217024 PMC10785363

[65] Millecamps M Lee S Foster DZ Stone LS. Disc degeneration spreads: long-term behavioural, histologic and radiologic consequences of a single-level disc injury in active and sedentary mice. Eur Spine J. (2021) 30:2238–46. 10.1007/s00586-021-06893-2 34216236

[66] Mi T Liu K Guo T Li L Wang Y Li C Analysis of the eighth intron polymorphism of NR6A1 gene in sheep and its correlation with lumbar spine number. Anim Biotechnol. (2023) 34(2):218–24. 10.1080/10495398.2021.1954529 34346290

[67] Wilke HJ Kettler A Wenger KH Claes LE. Anatomy of the sheep spine and its comparison to the human spine. Anat Rec. (1997) 247:542–55. 10.1002/(SICI)1097-0185(199704)247:4<542::AID-AR13>3.0.CO;2-P 9096794

[68] Proks P Stehlik L Nyvltova I Necas A Vignoli M Jekl V. Vertebral formula and congenital abnormalities of the vertebral column in rabbits. Vet J. (2018) 236:80–8. 10.1016/j.tvjl.2018.04.016 29871755

[69] Lu M Xu S Lei ZX Lu D Cao W Huttula M Application of a novel porous tantalum implant in rabbit anterior lumbar spine fusion model: *in vitro* and *in vivo* experiments. Chin Med J. (2019) 132(1):51–62. 10.1097/CM9.0000000000000030 30628959 PMC6629310

[70] Nystrom EU Blomberg SG Buffington CW. Transmural pressure of epidural veins in the thoracic and lumbar spine of pigs. Anesthesiology. (1998) 89:449–55. 10.1097/00000542-199808000-00022 9710404

[71] Kettler A Liakos L Haegele B Wilke H-J. Are the spines of calf, pig and sheep suitable models for pre-clinical implant tests? Eur Spine J. (2007) 16:2186–92. 10.1007/s00586-007-0485-9 17721711 PMC2140126

[72] Bailey JF Fields AJ Ballatori A Cohen D Jain D Coughlin D The relationship between endplate pathology and patient-reported symptoms for chronic low back pain Depends on lumbar paraspinal muscle quality. Spine. (2019) 44(14):1010–7. 10.1097/BRS.0000000000003035 30896590 PMC6597281

[73] Chiou S-Y Koutsos E Georgiou P Strutton PH. Association between spectral characteristics of paraspinal muscles and functional disability in patients with low back pain: a cohort study. BMJ Open. (2018) 8:e017091. 10.1136/bmjopen-2017-017091 29444776 PMC5829836

[74] Goubert D De Pauw R Meeus M Willems T Cagnie B Schouppe S Lumbar muscle structure and function in chronic versus recurrent low back pain: a cross-sectional study. Spine J. (2017) 17(9):1285–96. 10.1016/j.spinee.2017.04.025 28456669

[75] Becker S Bergamo F Schnake KJ Schreyer S Rembitzki IV Disselhorst-Klug C. The relationship between functionality and erector spinae activity in patients with specific low back pain during dynamic and static movements. Gait Posture. (2018) 66:208–13. 10.1016/j.gaitpost.2018.08.042 30205316

[76] Freeman MD Woodham MA Woodham AW. The role of the lumbar multifidus in chronic low back pain: a review. PMR. (2010) 2:142–6. 10.1016/j.pmrj.2009.11.006 20193941

[77] Laasonen EM. Atrophy of sacrospinal muscle groups in patients with chronic, diffusely radiating lumbar back pain. Neuroradiology. (1984) 26:9–13. 10.1007/BF00328195 6234476

[78] Chen X Hodges PW James G Diwan AD. Do markers of inflammation and/or muscle regeneration in lumbar Multifidus muscle and fat differ between individuals with good or poor outcome following microdiscectomy for lumbar disc herniation? Spine (Phila Pa 1976). (2021) 46:678–86. 10.1097/BRS.0000000000003863 33290379

[79] Methenitis S Spengos K Zaras N Stasinaki AN Papadimas G Karampatsos G Fiber type composition and rate of force development in endurance- and resistance-trained individuals. J Strength Cond Res. (2019) 33(9):2388–97. 10.1519/JSC.0000000000002150 28737590

[80] Cagnie B Dhooge F Schumacher C De Meulemeester K Petrovic M van Oosterwijck J Fiber typing of the erector spinae and multifidus muscles in healthy controls and back pain patients: a systematic literature review. J Manipulative Physiol Ther. (2015) 38(9):653–63. 10.1016/j.jmpt.2015.10.004 26547762

[81] Lee SH Park SW Kim YB Nam TK Lee YS. The fatty degeneration of lumbar paraspinal muscles on computed tomography scan according to age and disc level. Spine J. (2017) 17:81–7. 10.1016/j.spinee.2016.08.001 27497888

[82] Shi L Yan B Jiao Y Chen Z Zheng Y Lin Y Correlation between the fatty infiltration of paraspinal muscles and disc degeneration and the underlying mechanism. BMC Musculoskelet Disord. (2022) 23(1):509. 10.1186/s12891-022-05466-8 35637476 PMC9150320

[83] Teichtahl AJ Urquhart DM Wang Y Wluka AE Wijethilake P O’Sullivan R Fat infiltration of paraspinal muscles is associated with low back pain, disability, and structural abnormalities in community-based adults. Spine J. (2015) 15(7):1593–601. 10.1016/j.spinee.2015.03.039 25828477

[84] Teichtahl AJ Urquhart DM Wang Y Wluka AE O’Sullivan R Jones G Lumbar disc degeneration is associated with modic change and high paraspinal fat content - a 3.0 T magnetic resonance imaging study. BMC Musculoskelet Disord. (2016) 17(1):439. 10.1186/s12891-016-1297-z 27765024 PMC5073831

[85] Schiaffino S Reggiani C. Fiber types in mammalian skeletal muscles. Physiol Rev. (2011) 91:1447–531. 10.1152/physrev.00031.2010 22013216

[86] Blaauw B Schiaffino S Reggiani C. Mechanisms modulating skeletal muscle phenotype. Compr Physiol. (2013) 3:1645–87. 10.1002/cphy.c130009 24265241

[87] Qaisar R Bhaskaran S Van Remmen H. Muscle fiber type diversification during exercise and regeneration. Free Radic Biol Med. (2016) 98:56–67. 10.1016/j.freeradbiomed.2016.03.025 27032709

[88] Yokoyama Y. Analyses on the fiber compositions of the lumbar back muscles in mammals. Nippon Seikeigeka Gakkai Zasshi. (1982) 56:579–94. 7175293

[89] Neufuss J Hesse B Thorpe SK Vereecke EE D'Aout K Fischer MS Fibre type composition in the lumbar perivertebral muscles of primates: implications for the evolution of orthogrady in hominoids. J Anat. (2014) 224(2):113–31. 10.1111/joa.12130 24433382 PMC3969056

[90] Carlson H. Histochemical fiber composition of lumbar back muscles in the cat. Acta Physiol Scand. (1978) 103:198–209. 10.1111/j.1748-1716.1978.tb06207.x 150195

[91] Schmalbruch H Kamieniecka Z. Histochemical fiber typing and staining intensity in cat and rat muscles. J Histochem Cytochem. (1975) 23:395–401. 10.1177/23.6.125297 125297

[92] Dietrich J Handschuh S Steidl R Böhler A Forstenpointner G Egerbacher M Muscle fibre architecture of thoracic and lumbar Longissimus dorsi muscle in the horse. Animals. (2021) 11(3):915. 10.3390/ani11030915 33806991 PMC8004997

[93] Shahidi B Hubbard JC Gibbons MC Ruoss S Zlomislic V Allen RT Lumbar multifidus muscle degenerates in individuals with chronic degenerative lumbar spine pathology. J Orthop Res. (2017) 35(12):2700–6. 10.1002/jor.23597 28480978 PMC5677570

[94] Hesse B Fischer MS Schilling N. Distribution pattern of muscle fiber types in the perivertebral musculature of two different sized species of mice. Anat Rec (Hoboken). (2010) 293:446–63. 10.1002/ar.21090 20169566

[95] Ramos LAV França FJR Callegari B Burke TN Magalhães MO Marques AP. Are lumbar multifidus fatigue and transversus abdominis activation similar in patients with lumbar disc herniation and healthy controls? A case control study. Eur Spine J. (2016) 25(5):1435–42. 10.1007/s00586-015-4375-2 26769037

[96] Mannion AF Weber BR Dvorak J Grob D Müntener M. Fibre type characteristics of the lumbar paraspinal muscles in normal healthy subjects and in patients with low back pain. J Orthop Res. (1997) 15:881–7. 10.1002/jor.1100150614 9497814

[97] James G Sluka KA Blomster L Hall L Schmid AB Shu CC Macrophage polarization contributes to local inflammation and structural change in the multifidus muscle after intervertebral disc injury. Eur Spine J. (2018) 27(8):1744–56. 10.1007/s00586-018-5652-7 29948327

[98] James G Blomster L Hall L Schmid AB Shu CC Little CB Mesenchymal stem cell treatment of intervertebral disc lesion prevents fatty infiltration and fibrosis of the Multifidus muscle, but not cytokine and muscle fiber changes. Spine. (2016) 41(15):1208–17. 10.1097/BRS.0000000000001669 27135642

[99] Leimer EM Gayoso MG Jing L Tang SY Gupta MC Setton LA. Behavioral compensations and neuronal remodeling in a rodent model of chronic intervertebral disc degeneration. Sci Rep. (2019) 9(1):3759. 10.1038/s41598-019-39657-6 30842475 PMC6403208

[100] Wang J Zhu P Pan X Yang J Wang S Wang W Correlation between motor behavior and age-related intervertebral disc degeneration in cynomolgus monkeys. JOR spine. (2022) 5(1):e1183. 10.1002/jsp2.1183 35386757 PMC8966873

[101] Jung WW Kim HS Shon JR Lee M Lee SH Sul D Intervertebral disc degeneration-induced expression of pain-related molecules: glial cell-derived neurotropic factor as a key factor. J Neurosurg Anesthesiol. (2011) 23(4):329–34. 10.1097/ANA.0b013e318220f033 21659885

[102] Mosley GE Evashwick-Rogler TW Lai A Iatridis JC. Looking beyond the intervertebral disc: the need for behavioral assays in models of discogenic pain. Ann N Y Acad Sci. (2017) 1409:51–66. 10.1111/nyas.13429 28797134 PMC5730458

[103] Huang Y Lei L Zhu J Zheng J Li Z Wang H Pain behavior and phenotype in a modified anterior lumbar disc puncture mouse model. JOR spine. (2024) 7(1):e1284. 10.1002/jsp2.1284 38249720 PMC10797215

[104] Wawrose RA Couch BK Dombrowski M Chen SR Oyekan A Dong Q Percutaneous lumbar annular puncture: a rat model to study intervertebral disc degeneration and pain-related behavior. JOR spine. (2022) 5(2):e1202. 10.1002/jsp2.1202 35783914 PMC9238283

