# Supplementary material for: Structural changes of the multifidus in animal models of intervertebral disk degeneration: a systematic review
Source: Front Surg. 2024 Dec 16;11:1482821. doi: 10.3389/fsurg.2024.1482821 (PMC11685752; doi:10.3389/fsurg.2024.1482821)
Supplement: Supplementary file 2 [file Table2.docx]

**PubMed：**

(("Intervertebral Disc Degeneration"[Mesh] OR "Degeneration, Intervertebral Disc" OR "Disc Degeneration, Intervertebral" OR "Intervertebral Disc Degeneration*" OR "Disc Degeneration*" OR "Degeneration, Disc" OR "Degeneration, Intervertebral Disk" OR "Disk Degeneration, Intervertebral" OR "Intervertebral Disk Degenerations" OR "Disk Degeneration*" OR "Degeneration, Disk" OR "Disk Degradation*" OR "Degradation, Disk" OR "Degenerative Disc Disease*" OR "Disc Disease, Degenerative" OR "Disc Degradation*" OR "Degradation, Disc" OR "Degenerative Intervertebral Disc*" OR "Intervertebral Disc, Degenerative" OR "Degenerative Intervertebral Disk*" OR "Intervertebral Disk, Degenerative") AND ("Paraspinal Muscles"[Mesh] OR "Muscle, Paraspinal" OR "Muscles, Paraspinal" OR "Paraspinal Muscle" OR "Deep Muscles of the Back" OR "Intrinsic Muscles of the Back" OR Multifidus)) AND ("Models, Animal"[Mesh] OR "Animal Model*" OR "Model, Animal" OR "Laboratory Animal Model*" OR "Animal Model, Laboratory" OR "Animal Models, Laboratory" OR "Model, Laboratory Animal" OR "Experimental Animal Model*" OR "Animal Model, Experimental" OR "Animal Models, Experimental" OR "Model, Experimental Animal" OR "Models, Experimental Animal").

A search in November 2023 yielded 14 articles. No filters were used.

**Excerpta Medica (EMBASE)：**

(('intervertebral disk degeneration'/exp OR 'chondrosis, intervertebral' OR 'degeneration, intervertebral disk' OR 'degenerative disc disease' OR 'degenerative disc pathology' OR 'degenerative disk disease' OR 'degenerative disk pathology' OR 'degenerative inter-vertebral disc disease' OR 'degenerative intervertebral disc disease' OR 'degenerative intervertebral disk disease' OR 'degenerative vertebral disc disease' OR 'degenerative vertebral disk disease' OR 'disc degeneration' OR 'disc degenerative disease' OR 'discopathy' OR 'disk degeneration' OR 'disk degenerative disease' OR 'diskopathy' OR 'inter-vertebral disc degeneration' OR 'inter-vertebral disk degeneration' OR 'intervertebral chondrosis' OR 'intervertebral DD' OR 'intervertebral degenerative disc disease' OR 'intervertebral degenerative disk disease' OR 'intervertebral disc degeneration' OR 'intervertebral disc degenerative disease' OR 'intervertebral disk degenerative disease' OR 'intervertebral disk, degeneration' OR 'spinal DDD' OR 'spine DD' OR 'spine DDD' OR 'spondylochondrosis' OR 'vertebral DD' OR 'vertebral disc degeneration' OR 'vertebral disk degeneration' OR 'intervertebral disk degeneration') AND ('paraspinal muscle'/exp OR 'muscle, paraspinal' OR 'para spinal muscle' OR 'para vertrebral muscle' OR 'paraspinal muscles' OR 'paravertebral muscle' OR 'paraspinal muscle')) AND ('animal model'/exp OR 'animal disease model' OR 'animal models' OR 'model, animal' OR 'models, animal' OR 'animal model').

A search in November 2023 yielded 16 articles. No filters were used.

**Web of Science (Core Collection)：**

TS=(‘Intervertebral Dis? Degeneration*’ OR ‘Degeneration, Intervertebral Dis?’ OR ‘Dis? Degeneration, Intervertebral’ OR ‘Dis? Degeneration*’ OR ‘Degeneration, Dis?’ OR ‘Dis? Degradation*’ OR ‘Degradation, Dis?’ OR ‘Degenerative Disc Disease*’ OR ‘Disc Disease, Degenerative’ OR ‘Degenerative Intervertebral Dis?*’ OR ‘Intervertebral Dis?, Degenerative’) AND TS=(‘Paraspinal Muscle*’ OR ‘Muscle*, Paraspinal’ OR ‘Deep Muscles of the Back’ OR ‘Intrinsic Muscles of the Back’ OR Multifidus) AND TS=(‘Model*, Animal’ OR ‘Animal Model*’ OR ‘Laboratory Animal Model*’ OR ‘Animal Model*, Laboratory’ OR ‘Model, Laboratory Animal’ OR ‘Experimental Animal Model*’ OR ‘Animal Model*, Experimental’ OR ‘Model*, Experimental Animal’)

A search in November 2023 yielded 46 articles. No filters were used.

**Cochrane Library：**

A search in November 2023 yielded 0 articles. No filters were used.

**MEDLINE Ovid：**

(("Degeneration, Intervertebral Disc" OR "Disc Degeneration, Intervertebral" OR "Intervertebral Disc Degeneration*" OR "Disc Degeneration*" OR "Degeneration, Disc" OR "Degeneration, Intervertebral Disk" OR "Disk Degeneration, Intervertebral" OR "Intervertebral Disk Degenerations" OR "Disk Degeneration*" OR "Degeneration, Disk" OR "Disk Degradation*" OR "Degradation, Disk" OR "Degenerative Disc Disease*" OR "Disc Disease, Degenerative" OR "Disc Degradation*" OR "Degradation, Disc" OR "Degenerative Intervertebral Disc*" OR "Intervertebral Disc, Degenerative" OR "Degenerative Intervertebral Disk*" OR "Intervertebral Disk, Degenerative") AND ("Paraspinal Muscle*" OR "Muscle*, Paraspinal" OR "Deep Muscles of the Back" OR "Intrinsic Muscles of the Back" OR Multifidus)) AND ("Model*, Animal" OR "Animal Model*" OR "Laboratory Animal Model*" OR "Animal Model*, Laboratory" OR "Model, Laboratory Animal" OR "Experimental Animal Model*" OR "Animal Model*, Experimental" OR "Model*, Experimental Animal")

A search in November 2023 yielded 13 articles. No filters were used.
