# Supplementary figures and images for: Structural changes of the multifidus in animal models of intervertebral disk degeneration: a systematic review
Source: Front Surg. 2024 Dec 16;11:1482821. doi: 10.3389/fsurg.2024.1482821 (PMC11685752; doi:10.3389/fsurg.2024.1482821)

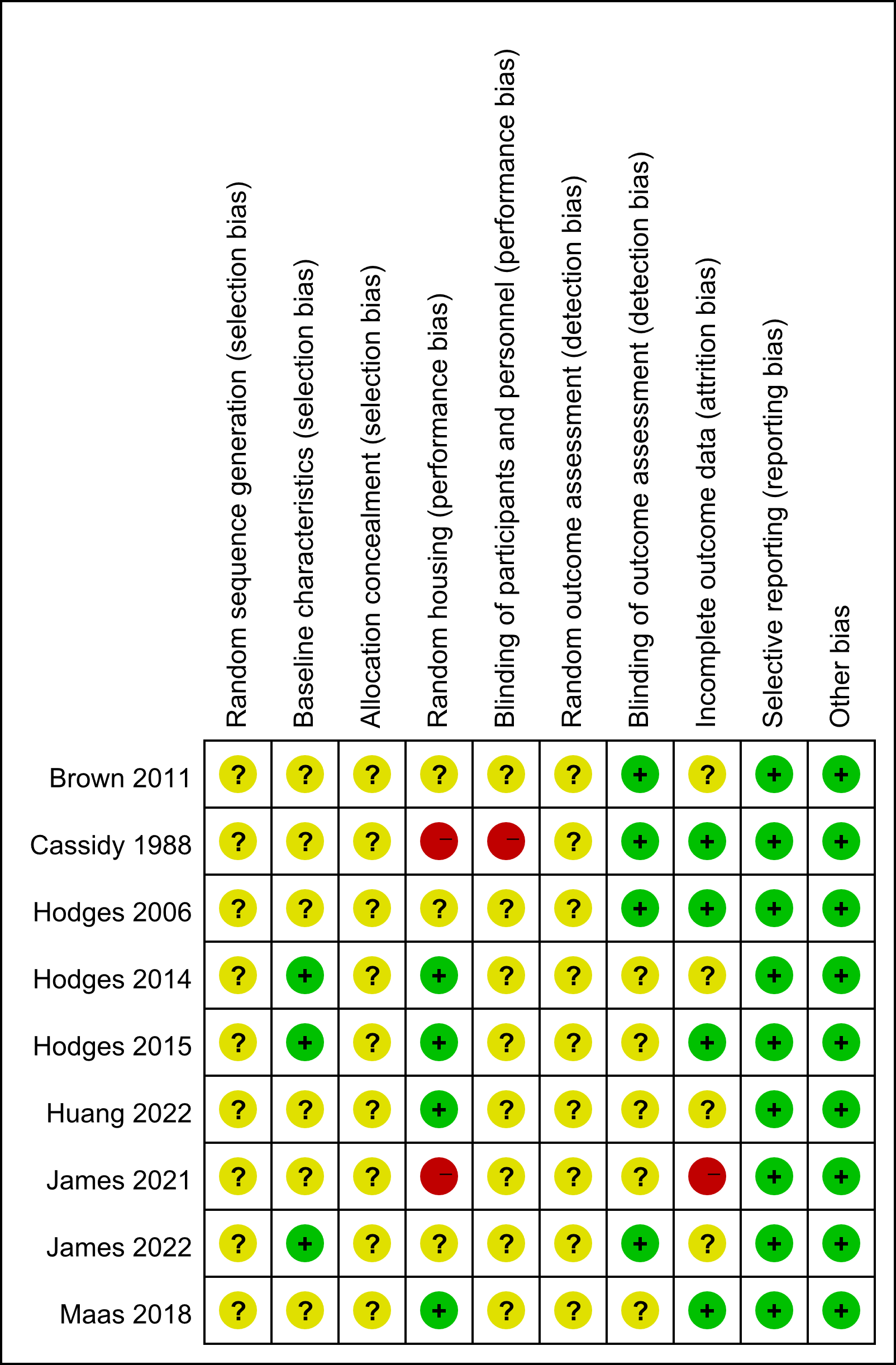


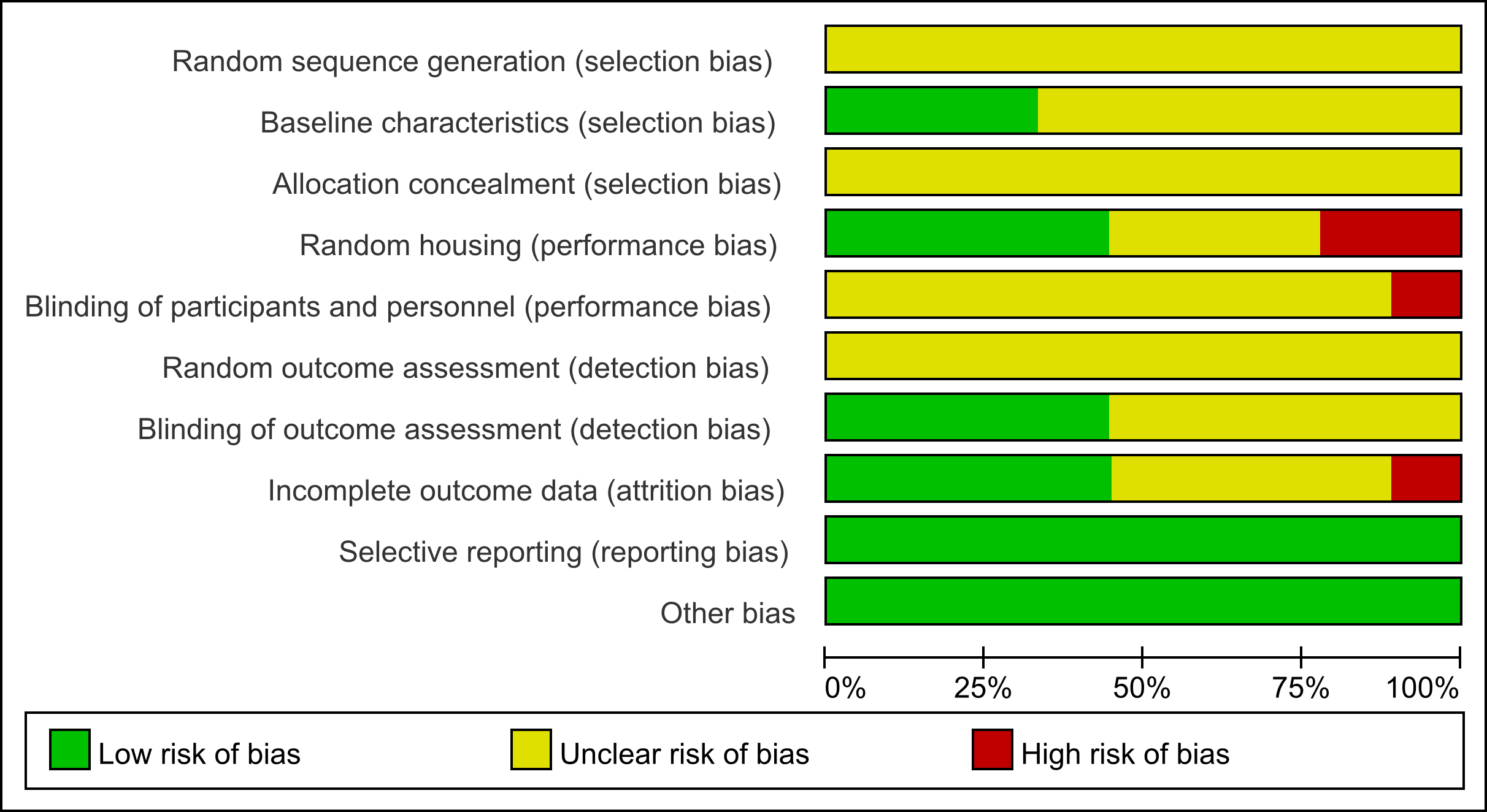

Supplement: Supplementary file 4 [file Table4.docx]
