# Supplementary material for: Structural changes of the multifidus in animal models of intervertebral disk degeneration: a systematic review
Source: Front Surg. 2024 Dec 16;11:1482821. doi: 10.3389/fsurg.2024.1482821 (PMC11685752; doi:10.3389/fsurg.2024.1482821)
Supplement: Supplementary file 5 [file Table5.docx]

**Identification of studies via other methods**

**Identification of studies via databases and registers**

**Records identified (total=1)** from other methods:

Websites (n = 0)

Organisations (n = 0)

Citation searching (n = 1)

**Records removed *before screening*:**

Duplicate records removed using Zotero

(n = 34)

Records marked as ineligible by automation tools (n = 0)

Records removed for other reasons (n = 0)

**Records identified** **(total = 89)** from:

**Databases (n = 89):**

PubMed (n = 14)

EMBASE (n = 16)

Web of Science (n = 46)

Cochrane Library (n = 0)

MEDLINE Ovid (n = 13)

**Registers (n = 0)**

**Identification**

Records screened for title and abstract by two independent reviewers (n = 55)

Records excluded on consensus by two reviewers (n = 36)

Reports assessed for eligibility

(n = 1)

Reports excluded:

(n = 0)

Reports sought for retrieval

(n = 1)

Reports not retrieved

(n = 0)

Reports / studies sought for retrieval for full text screening (n = 19)

Reports not retrieved (n = 0)

**Screening**

**Reports excluded reasons (total = 11):**

Study not in English language (n = 2)

Study not established an animal model of disc degeneration (n = 1)

Studies with none of the following outcomes: Mass, CSA, FI, Stiffness, Fibre type composition, or fibre size of multifidus (n = 5)

Studies not directly secondary to MF structural changes in IVDD animal models (with additional interventions) (n = 2)

Retracted (n = 1)

Reports / studies assessed for eligibility (n = 19)

Studies included in review

(n = 9)

Reports of included studies

(n = 0)

**Included**

*From:*  Page MJ, McKenzie JE, Bossuyt PM, Boutron I, Hoffmann TC, Mulrow CD, et al. The PRISMA 2020 statement: an updated guideline for reporting systematic reviews. BMJ 2021;372:n71. doi: 10.1136/bmj.n71. For more information, visit: <http://www.prisma-statement.org/>
